# Effects of silica soil amendment against *Exserohilum rostratum,* the fungal pathogen of rice brown spot disease in Peninsular Malaysia

**DOI:** 10.1038/s41598-022-19308-z

**Published:** 2022-09-20

**Authors:** Ainu-Shahirah Mahmad-Toher, Nisha Govender, Deivaseeno Dorairaj, Mui-Yun Wong

**Affiliations:** 1 Department of Plant Protection, Faculty of Agriculture, Universiti Putra Malaysia (UPM), 43400 Serdang, Selangor Malaysia; 2 Institute of Systems Biology (INBIOSIS), Universiti Kebangsaan Malaysia (UKM), 43600 Bangi, Selangor Malaysia; 3 Institute for Environment and Development (LESTARI), Universiti Kebangsaan Malaysia (UKM), 43600 Bangi, Selangor Malaysia; 4 Institute of Plantation Studies, Universiti Putra Malaysia (UPM), 43400 Serdang, Selangor Malaysia

**Keywords:** Microbiology, Molecular biology, Plant sciences

## Abstract

Rice brown spot (BS) exerts devastating agronomic effects on grain quality and overall productivity. In Peninsular Malaysia, BS disease incidence is fairly prevalent and little is known about the diversity of BS pathogens in the local granaries. Fifteen isolates from BS symptomatic rice plants were identified at five different rice granaries across Peninsular Malaysia. Based on the morphological and molecular analyses, two isolates were confirmed as *Bipolaris oryzae* while the rest were identified as *Exserohilum rostratum*. Phylogenetic tree analysis revealed that BS incidence in rice granaries in Peninsular Malaysia is caused by a pair of closely related fungal pathogens, *E. rostratum* and *B. oryzae,* with the former being more predominant. Cultural characterization of *E. rostratum* isolate KT831962 showed the best growth and sporulation activity on corn meal agar plates incubated in complete darkness. The effects of calcium silicate (CaSiO_3_) and rice husk ash (RHA) soil amendment against MR219 and MR253 rice varieties were evaluated during rice-*E. rostratum* interaction. Results showed that soil amelioration using CaSiO_3_ and RHA singly and in combination with manganese (Mn) significantly reduced rice BS disease severity. The BS disease index was reduced significantly to less than 31.6% in the silicon-treated rice plants relative to the control plants at 41.2%. Likewise, the grain yield at the harvest stage showed significantly higher yield in the Si-treated rice plants in comparison to the control, non-Si treated rice plants. The findings highlight the potential of RHA agro-waste as Si fertilizer in a sustainable rice production system.

## Introduction

The Asian region accounts for 4.4 billion people from 50 different countries^1^. Rice, *Oryza sativa* L., is the staple food for Asians and thus, a major portion of worldwide rice cultivation areas are found within the tropical region. The rice plant has a life span of three to six months depending on variety and climate. The cereal grass thrives under diverse ecosystems and the productivity ranges from about 1.5 to 8 metric tonne/hectare in countries such as Malaysia^2^.

Rice production is negatively affected by both biotic and abiotic stresses as well as farming and management practices. Amongst the biotic stressors, disease occurrences are highly influenced by climate and soil variation^3^. The tropical region has a warm and humid environment that is conducive and well-suited for the survival and propagation of phytopathogens^4,5^. Such an ideal environment favours rice diseases such as the blast, brown spot (BS), sheath blight and false smut. Amongst them, BS is one of the most prevalent and devastating diseases which affects rice production^6^. In Asia, rice BS caused by *Bipolaris oryzae* Shoemaker has been reported to reduce yield by up to 90%^7^. The notorious rice pathogen has been impacting the rice industry since the Bengal famine in 1943 wherein about 2–3 million people died of starvation due to stagnant agricultural productivity^8^. Various fungal species have been documented as the causal agents of BS with each producing different characteristic symptoms on host. For instance, brown leaf spot is caused by *B. oryzae*^9,10^, narrow brown leaf spot is caused by *Cercospora oryzae*^9,11^ and the leaf blast is caused by *Pyricularia oryzae*^12^. In Malaysia, rice BS reported causal pathogens are *B. oryzae*^13,14^, *Exserohilum rostratum*^15^ and *B. cactivora*^16^.

The occurrence of rice BS is implicated with a low nutrient condition^17^ especially potassium, manganese, silicon (Si), iron, calcium and magnesium^18,19^. Other contributing factors include high soil pH, low organic matter and low cation exchange capacity^20^. Most fungal-causing diseases are controlled by fungicides, nevertheless, in many disease control strategies, the application of nutrients such as Si and Zn were shown to significantly reduce plant disease incidence and severity^21,22^.

Rice is a well-known Si hyper-accumulator and Si accumulation in rice plants may exceed 10% of the shoot’s dry weight^23^. Hence, the exogenous application of Si yields a pronounced effect on stressed rice plants. In highly weathered tropical soils, the plant available Si is generally low due to desilication-aluminization^24^ and repeated mono-cropping with rice^6^. Correspondingly, the application of Si has been shown to lower the intensity of pests and diseases^25–27^, improve soil pH, enhance absorption of phosphate, sulphate and nitrate^22^, and improve tolerance to drought and plant resistance to metal toxicity^28^. Moreover, Si provides resistance to bacterial and fungal diseases^29,30^, low temperatures^31^, salinity^32,33^, aluminium toxicity^34^ besides influencing nitrogen and phosphorus availability in plant tissues^35,36^.

In disease control and biofertilization strategies, calcium silicate (CaSiO_3_) has been popularly employed as an ultimate Si source in comparison to other alternatives such as potassium silicate, rice husk ash (RHA) and rice stem ash. Application of CaSiO_3_ at 5000 kg/ha (Si at 1000 kg/ha) increased plant tissue Si content by 3–5%^37,38^ and is deemed a rather expensive Si source than agricultural waste-based alternatives such as RHA^39^.

Manganese (Mn) is an essential immobile micronutrient involved in photosynthesis, respiration, fatty acid and protein synthesis, as well as enzyme activation^40^. While Mn deficiency can decrease chlorophyll content, photosynthesis and change the activity of superoxide dismutase (SOD) and inhibit plant growth^41,42^, its toxicity leads to brown spotting of mature leaves^43^. Si reportedly alleviates Mn toxicity in many plants including rice which is one of the most Mn-tolerant crops^44–49^. According to Yoshida^50^, high Mn in rice tissues could increase yield. Based on the plant species, variety and environment, the optimum Mn concentration varies between 30 to 500 mg kg^−1^ while deficiency and toxicity range between 10 to 20 mg kg^−1^ and 200 to 5300 mg kg^−1^, respectively^51,52^. In rice, Mn deficiency takes place when the plant tissue concentration hits below 20 mg kg^−1^. In contrast, the maximum Mn tolerance level could hike up to 2500 mg kg^−1^^50^.

In this study, the BS pathogens are isolated at five important rice granaries across Peninsular Malaysia (high and low-performing granaries) and the most dominant causal pathogen is determined via morphological and molecular analyses. The cultural characterization of the most virulent BS causal pathogen is presented. Further, the effects of two silicon sources namely RHA and CaSiO_3_ during host–pathogen interaction were evaluated by the BS disease severity index and grain yield scored at the rice harvest stage.

## Materials and methods

### Isolation of fungal pathogens: location and sample procurement

**Figure 1:**
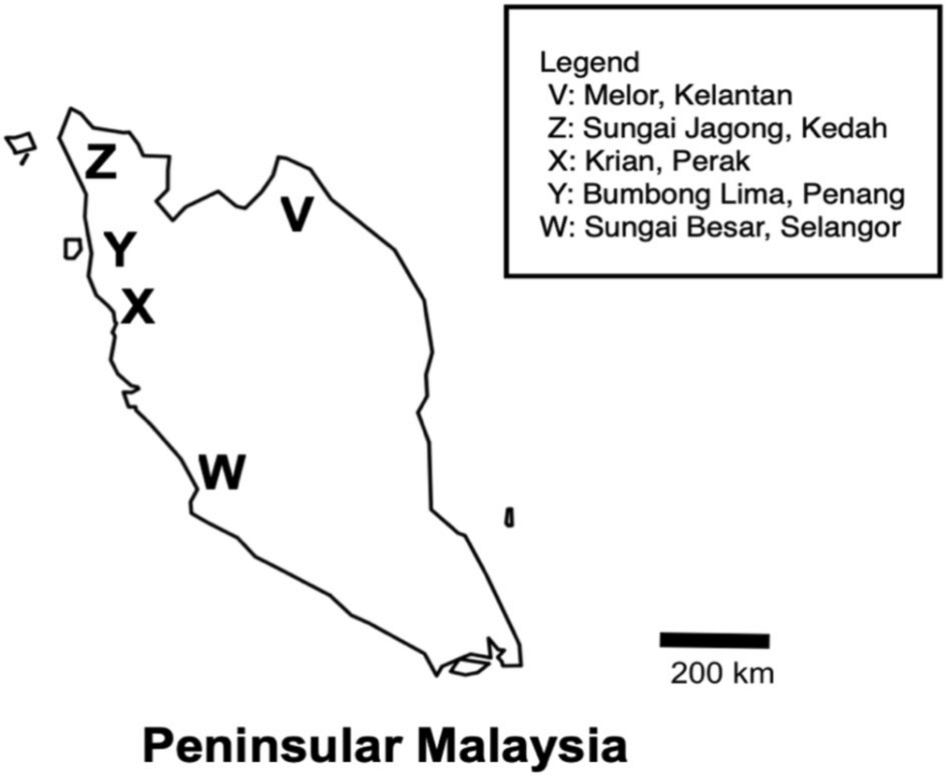
Sampling for brown spot infected rice plants at five different rice granaries in Peninsular Malaysia. Sampling locations (V, W, X, Y and Z) are indicated as area followed by state names. W, Y and Z are high performing (> 4 tonnes/ha) granaries whilst V and X are low performing (< 4 tonnes/ha) granaries (Khazanah Research Institute, 2019).

Brown spot (BS) symptoms were observed on leaves and glumes parts of the plants available in rice granaries located in five different states in Peninsular Malaysia: Melor (Kelantan), Sungai Jagong (Kedah), Bumbong Lima (Penang), Krian (Perak) and Sungai Besar (Selangor) during the growing seasons of November 2012-December 2013 (Fig. 1). All sampling activities performed in the study complied with the Department of Agriculture, Malaysia’s guidelines and legislation. Disease plants showing BS characteristic symptoms were collected at the near harvesting stage (about 80–100 days from sowing), put into plastic bags and transported to the Laboratory of Mycology, Department of Plant Protection, Universiti Putra Malaysia for further analysis. The samples were excised into cubes of 1 × 1 cm, upon surface sterilization with 10% sodium hypochlorite solution and then rinsed in sterilized distilled water^53^. Each leaf cube was then plated onto potato dextrose agar medium (PDA) (Difco, USA) and incubated for 10 days at 28 ± 2 °C in a diurnal cycle of alternate dark and light (fluorescent light) conditions that mimic 12 h light and dark photoperiod^54^. The growing mycelium was transferred onto fresh PDA plates. The resultant pure cultures obtained were subjected to morphological and molecular identifications.

### Morphological characterization of BS causal agents

For the morphological identification of BS causal agents, the mycelia, conidia and conidiophores of each isolate (10-day-old) were collected and observed under a compound microscope (Olympus, USA) according to Sivanesan et al.^55^ and Cardona and Gonzalez^56^. Each isolate was screened visually for colour, shape and size.

### Molecular characterization of fungal pathogens

Mycelium (10-day-old isolate) was harvested and ground into a fine powder with liquid nitrogen. Total DNA was isolated using Qiagen DNeasy® Mini Plant Kit according to the manufacturer’s protocol. The quality and quantity of the DNA were determined by MultiScanGo microplate spectrophotometer (Thermo Scientific, USA). Universal primers corresponding to the fungal ITS region of 500–600 bp (expected amplicon size) were designed according to White et al.^57^: forward primer; 5′-TCCTCCGCTTATTGATATGC-3′ and reverse primer; 5′-GAAGTAAAAGTCGTAACAAGG-3’. A PCR reaction was performed according to the DreamTaq Green PCR Master Mix (2X) (Thermo Scientific, USA) manufacturer’s instructions: 40 ng of gDNA, 12.5 µl of DreamTaq Green PCR Master Mix (2X), 4 mM MgCl_2_, 2 µM of each primer in a final volume of 25 µl. Amplifications were run on a Thermocycler system (C1000 Touch, BIORAD, USA) under the following conditions: denaturation at 95 °C for 3 min, followed by 35 cycles of denaturation at 95 °C for 30 s, annealing at 56 °C for 30 s, extension at 72 °C for 1 min and a final extension for 10 min at 72 °C. All amplicons obtained were sequenced by First Base Laboratory Sdn. Bhd. Malaysia. For every fungal isolate described, a total of three biological replicates were used with two technical replicates.

### Phylogenetic analysis

The amplicon sequence corresponding to the fungal ITS regions from all the 20 isolates were subjected to sequence similarity search using the Basic Local Alignment Search Tool (BLAST) available at National Center for Biotechnology Information (NCBI) website. An evolutionary analysis of the putative sequence (from 20 isolates), *Exserohilum rostratum* (GQ179755.1, GQ169762, GQ478868.1), *Bipolaris oryzae* (JX256416 and KJ922383.1) and *Lectosphaeria bicolor* (outlier) sequences were aligned by MEGA6^58^ and a phylogenetic tree was inferred using the CLUSTALW BioEdit version 7.0.5^59^. The evolutionary relationship among the putative target isolates was determined by the Neighbour-Joining method^60^ and the evolutionary distances were computed by the distance method^61^.

### Pathogenicity test

For all the isolates obtained, a pathogenicity test was conducted according to Cardona and Gonzalez^56^ with slight modifications. Leaves were detached from rice plants (MR219 variety) at the growth stages of vegetative [30 days after planting (DAP)], reproductive (60 DAP) and harvest (100 DAP). The leaves (5 × 5 cm) were surface-sterilized with 10% alcohol and placed on a moist paper towel in a Petri dish^62^. A mycelium plug (1 × 1 cm) from each isolate was inoculated onto the leaves and incubated at 28 ± 2 °C for 10 days. The inoculated leaf samples were then screened for brown spot characteristic symptoms and the visual rating was scored accordingly^63^.

### Cultural characterization of the most virulent fungal isolate

The most virulent isolate (as determined from the pathogenicity test) was inoculated onto different growth media and conditions as well as further incubated to determine sporulation activity. Each treatment was represented by six biological replicates. Mycelial plugs (1 × 1 cm) from the peripheral region of a 4-day-old primary culture were inoculated on the following media: potato dextrose agar (PDA) (Eur. Pharma, CONDA Lab, Madrid), malt extract agar (MEA) (Eur. Pharma, CONDA Lab, Madrid), corn meal agar (CMA) (Eur. Pharma, CONDA Lab, Madrid) and Czapex-dox agar (CA) (Eur. Pharma, CONDA Lab, Madrid). All plates were incubated under the following light cycles: T1; 12 h fluorescent light + 12 h black light, T2; 12 h fluorescent light + 12 h complete darkness, T3; 12 h black light + 12 h complete darkness, and T4; 24 h darkness. At day-7-post-inoculation the mycelium diameter was measured using a ruler and the best growth plates were placed in a moisture chamber for a week. Following incubation on day-14-post-inoculation, spores were collected by suspending 10 ml of sterile distilled water onto the dense mycelium followed by a gentle scraping using a sterilized L-shaped glass rod. The number of spores formed on the plate was calculated using a haemacytometer (Neubauer Improved, Germany)^64^.

### Plant establishment

Three independent pot experiments were performed at Ladang 2 Greenhouse Complex, Faculty of Agriculture, Universiti Putra Malaysia. All experiments were conducted between September 2013 and December 2014 under the following environmental conditions: humidity = 90%, average minimum temperature = 22.6 °C and average maximum temperature = 33.8 °C.

Rice seeds from the MR219 and MR253 varieties were purchased from Malaysian Agricultural Research and Development Institute (MARDI). MR219 used to be grown in 90% of rice granaries in Malaysia^65^ since its introduction in 2001 but over time it became susceptible to blast disease. Hence, MR253 which was an improved variety with moderate resistance to blast disease was introduced. Seeds of both varieties were immersed in distilled water for 2 days and the drained seeds were placed onto a moist filter paper in a large Petri dish. Upon the visible formation of radicles (germination), a total of 4 seedlings were sown into each potting medium. Silty clay soil procured from a rice field located at Sawah Sempadan, Tanjong Karang were sterilized using a 2% formaldehyde solution as described by Miller^66^. Each polyethene container (40 cm × 28 cm) was filled with 15 kg of sterilized and air-dried soil. The soil type was silty clay soil classified as fine textured by USDA Soil Taxonomy System (pH 4.62, clay 50.44%, silt 41.66%, and sand 7.90%, cation exchanged capacity = 1.61 cmol_c_kg^−1^) and the elemental composition is described as following: Al:0.16 mg kg^−1^, Cu:0.16 mg kg^−1^, Zn:1.28 mg kg^−1^, Fe:41.61 mg kg^−1^,Mn:4.43 mg kg^−1^ and Si;5.33 mg kg^−1^.

### Experimental design and treatments

Two rice varieties, MR219 and MR253 were tested against five treatments. Each treatment (T1-T5) condition represented by four biological replicates is described as following: T1; control, T2; RHA, T3; RHA +Mn, T4; CaSiO_3_, T5; CaSiO_3_ +Mn. The experiment was arranged in a randomized complete block design (RCBD). Rice husk ash (RHA) and calcium silicate (CaSiO_3_)(Si 24%) were purchased from Padi Beras Nasional Berhad (BERNAS), Malaysia and Kaolin Sdn. Bhd., Malaysia, respectively. The individual components of RHA, CaSiO_3_ and Mn chelate were applied at 700 kg/ha, 1500 kg/h, and 30 kg/ha, respectively by mixing them in the soil. All rice plants were supplied with 120 kg/ha nitrogen (N) in the form of urea in three split applications at 15, 35 and 55 days after planting (DAP). Phosphorus (P) was applied in the form of super triphosphate at a rate of 70 kg/ha while potassium (K) was applied at 80 kg/ha as a basal dressing on day 55 before the panicle initiation (PI) stage in the form of muriate of potash^67^.

### Artificial inoculation of rice plants with *Exserohilum rostratum* inoculum

The *E. rostratum* AS.P2 isolate was selected for large-scale inoculums preparation. The morphological and molecular description of *E. rostratum* AS.P2 is described in our previous study^15^. A 10-day-old pure culture was inoculated onto corn meal agar (CMA) and the plate was incubated at 28 ± 2 °C for 2 weeks. Spores were collected from each plate as described previously and the concentration of spores was adjusted to 5 × 10^3^ spore/ml^64^.

At 40 DAP, five fully mature leaves from each experimental pot were randomly selected. About 10 ml of freshly collected *E. rostratum* spore suspension (final concentration at 5 × 10^3^ spore/ml) was applied on each leaf surface using a syringe until run-off. At the harvest stage, the BS disease symptoms were visually observed on the grains following a scale allotted manually: 0-discoloration free (no symptom), 1 = 40% grain browning 2 = 40–80% grain browning, 3 = 40–80% grain blackening, and 4 = 100% grain browning/blackening. The BS severity index (BSI) and grain yield (GY) were scored for each experimental pot The first was measured according to McKinney^68^ with slight modifications. All results were expressed as average values of three independent experiments.

$${\text{Brown}}\,{\text{spot}}\,{\text{index}}\,({\text{BSI}}) = [ (r \times nr)/(R \times Nr)] \times 100$$

where *r*, scale score of infected grain; *nr*, total number of infected grains/experimental pot; *R*, highest scale score of the infected grain; *Nr*, total number of grains/experimental pot.

$${\text{Grain}}\,{\text{yield}}\,({\text{GY}}) = [{ (}n - nr{)}/n] \times 100$$

*n*, total number of filled grains/experimental pot; *nr*, total number of infected grains/experimental pot.

#### Statistical analysis

The average data from three independent pot experiments were analysed using SAS program (version 9.2) (SAS Institute, North Carolina, USA). The differences among treatments were determined using the least significant difference (LSD) test at p ≤ 0.05.

## Results

### Identification of brown spot causal pathogens in Peninsular Malaysia

**Figure 2:**
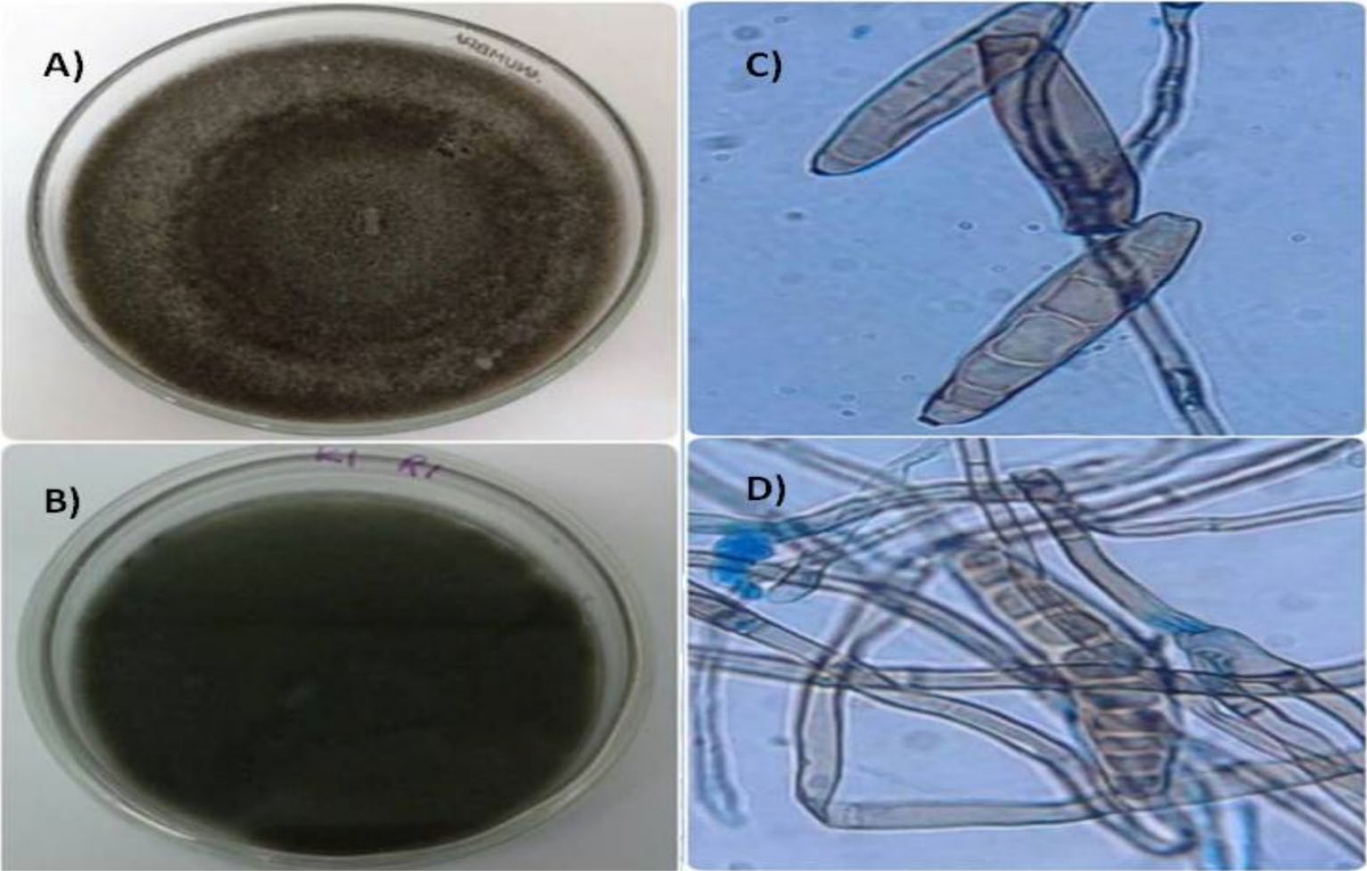
Morphological view of rice brown spot fungal pathogens: *Exserohilum rostratum* and *Bipolaris oryzae* axenic cultures and under light microscope 100× magnification. (**A**) 14-day-old *E. rostratum*; (**B**) 14-day-old *B. oryzae*; (**C**) *B. oryzae* conidia in light colour displaying minute curve and almost cylindrical shape; (**D**) *E. rostratum* conidia displaying darkened cylinder and protruding hilum.

In total, 15 fungal isolates were obtained from five different rice granaries located in Peninsular Malaysia. Based on the morphological analysis, the putative *E. rostratum* isolates showed a fluffy mycelium texture with full cotton-like confluence covering the entire petri dish on day 14 after inoculation (Fig. 2A). Similarly, the putative *B. oryzae* isolates showed confluent growth, however, free of cotton-like texture (Fig. 2B). Both fungal isolates (mycelium) were grey-to-dark grey. The conidiophores were dark brown or olivaceous cylindrical with an average thickness of 5–10 µm. It has septate (simple and geniculate type) and showed a pale discolouration towards the apex region. Putative *B. oryzae* showed a minute curve and almost cylindrical-shaped conidia (Fig. 2C). The putative *B. oryzae* descriptions corroborated with other previous studies^69,70^. The putative *E. rostratum* conidia showed a darker colouration towards the cylinder and protruding hilum (Fig. 2D), in agreement with Sivanesan^55^. The genus *Exserohilum* segregated from its closely related species, which previously accommodated *Bipolaris* or *Drechslera Ito sens.* Lat.^71,72^. The *Exserohilum* spp. has an apparent protruding conidial hilum which distinguishes it from its close relative species. *E. rostratum* produced dark brown conidiophores with protruding hilum, septate, simple, geniculate type and pale discolouration towards the apex region^55,56^.

**Figure 3:**
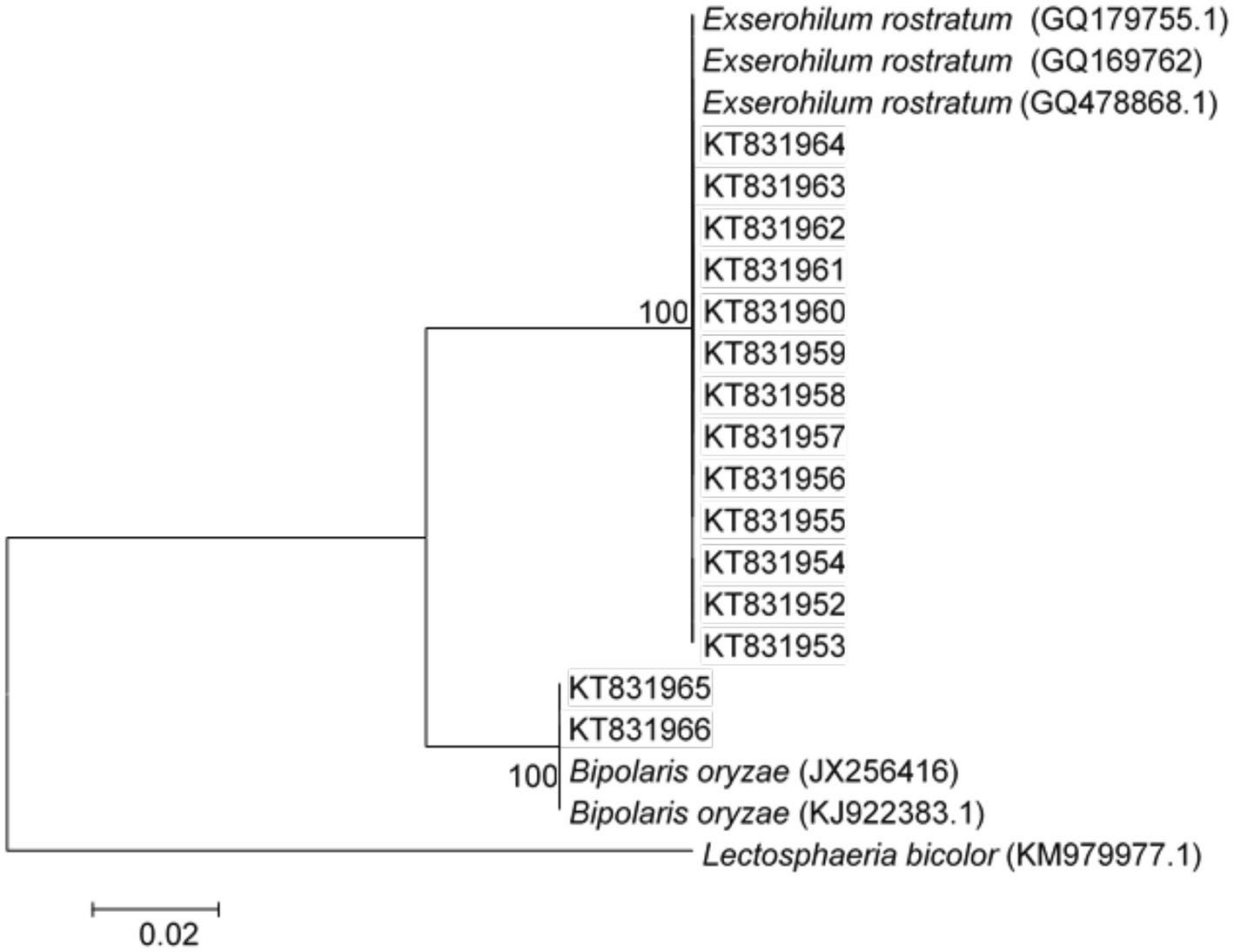
Phylogenetic analysis of rDNA-ITS region of fungal isolates obtained from brown spot diseased rice plants in Peninsular Malaysia. The evolutionary analysis of both *Exserohilum rostratum* and *Bipolaris oryzae* is inferred based on rDNA-ITS sequences using Neighbour-Joining method with 500 replications. Reference sequences including *E. rostratum* (GQ179755.1, GQ169762, GQ478868.1), and *B. oryzae* (JX256416 and KJ922383.1).

PCR amplification of the rDNA-ITS sequences of these putative isolates confirmed their identities. The rDNA-ITS sequence showed similarity to *E. rostratum* and *B. oryzae* at 99–100% similarity index. In Melor and Sungai Jagong rice granaries (located in the northern region of Peninsular Malaysia), both *B. oryzae* and *E. rostratum* were identified as BS causal agents. In contrast, only *E. rostratum* was identified at Krian, Sungai Besar and Bumbong Lima rice granaries. Four isolates were obtained in the Melor rice granary; three isolates of *E. rostratum* (AS.K1, AS.K3 and AS.K4) whilst only one isolate represented *B. oryzae* (K2). There were two different races of *E. rostratum* (AS.PR1 and AS.PR2) obtained from Krian. Four isolates (AS.B1, AS.B2, AS.B3 and AS.B4) and three isolates (AS.P1, AS.P2 and AS.P3) of *E. rostratum* were obtained in Sungai Besar and Bumbong Lima rice granaries, respectively. In Sungai Jagong rice granary, both *E. rostratum* (AS.KD2) and *B. oryzae* (KD1) were present with one isolate each (Table 1). The phylogenetic analysis showed two clades comprised of *E. rostratum* and *B. oryzae* at 95% bootstrap value. Thirteen *E. rostratum* isolates and two *B. oryzae* isolates were clustered with GenBank rDNA-ITS sequences (related species) at 100% bootstrap value. *Lectosphaeria bicolor* ITS sequence fed into the phylogenetic analysis presents an out-group (Fig. 3).

**Table 1:** Brown spot causal agents isolated from rice granaries in Peninsular Malaysia.

Fungal species	GeneBank Accession	GeneBank Reference	Isolate ID	Rice granary location
*E. rostratum*	KJ439663.1	KT831952	AS.K1	Melor, Kelantan
*B. oryzae*	GU480770.1	KT831965	K2	Melor, Kelantan
*E. rostratum*	KP340122.1	KT831953	AS.K3	Melor, Kelantan
*E. rostratum*	KP340122.1	KT831954	AS.K4	Melor, Kelantan
*E. rostratum*	KP340085.1	KT831955	AS.PR1	Krian, Perak
*E. rostratum*	KF897861.1	KT831956	AS.PR2	Krian, Perak
*E. rostratum*	KJ830935.1	KT831957	AS.B1	Sungai Besar, Selangor
*E. rostratum*	JX868670.1	KT831958	AS.B2	Sungai Besar, Selangor
*E. rostratum*	JX868670.1	KT831959	AS.B3	Sungai Besar, Selangor
*E. rostratum*	KP340122.1	KT831960	AS.B4	Sungai Besar, Selangor
*E. rostratum*	KJ887577.1	KT831961	AS.P1	Bumbong Lima, Penang
*E. rostratum*	KF897860.1	KT831962	AS.P2	Bumbong Lima, Penang
*E. rostratum*	KP340122.1	KT831963	AS.P3	Bumbong Lima, Penang
*B. oryzae*	HM572291.1	KT831966	KD1	Sungai Jagong, Kedah
*E. rostratum*	KP340122.1	KT831964	AS.KD2	Sungai Jagong, Kedah

The isolate and GeneBank Accession of the internal transcribed fungal (ITS) sequences are expressed at > 99% similarity index.

A pathogenicity test was conducted on all the 15 isolates to confirm that each isolate is a causal agent of BS on an individual evaluation. The artificial inoculation of fungal pathogens on leaves (detached from rice plants) showed orange-brown elliptical lesions at 3 to 6 DAI. The lesions turned greyish or whitish along with reddish brown margin as the disease progresses from 7–10 DAI. Generally, all isolates showed a disease index in between 3.75–5 (5, highest score). Three isolates, K2 (KT831965), AS.P2 (KT831962) and KD1 (KT831966) isolated from Melor, Bumbong Lima and Sungai Jagong obtained the highest rating value (5.00); super-virulent isolates (Table 2).

**Table 2:** Rating scores of rice brown spot fungal isolates, as determined using the leaf detach method.

Isolate	Species name	Location	Rating value
K2	*Bipolaris oryzae*	Melor, Kelantan	5.00
AS.P2	*Exserohilum rostratum*	Bumbong Lima, Penang	5.00
KD1	*Bipolaris oryzae*	Sungai Jagong, Kedah	5.00
AS.B2	*Exserohilum rostratum*	Sungai Besar, Selangor	4.75
AS.P1	*Exserohilum rostratum*	Bumbong Lima, Penang	4.75
AS.P3	*Exserohilum rostratum*	Bumbong Lima, Penang	4.75
AS.KD2	*Exserohilum rostratum*	Sungai Jagong, Kedah	4.75
AS.B3	*Exserohilum rostratum*	Sungai Besar, Selangor	4.50
AS.K4	*Exserohilum rostratum*	Melor, Kelantan	4.25
AS.PR2	*Exserohilum rostratum*	Krian, Perak	4.25
AS.B1	*Exserohilum rostratum*	Sungai Besar, Selangor	4.25
AS.K3	*Exserohilum rostratum*	Sungai Jagong, Kedah	4.00
AS.K1	*Exserohilum rostratum*	Melor, Kelantan	3.75
AS.PR1	*Exserohilum rostratum*	Krian, Perak	3.75
AS.B4	*Exserohilum rostratum*	Sungai Besar, Selangor	3.75

### Cultural characterization of *E. rostratum* virulent isolate

The *E. rostratum* AS.P2 (KT831962) virulent isolate was selected for cultural characterization. Growth parameter, the *E. rostratum* mycelium diameter (MD) measured at day-7-post-inoculation showed differences with growth media and conditions. Both CMA and CA under the various growth conditions (T1-T4) showed the greatest *E. rostratum* MD at 7.70 cm, followed by PDA and MEA at *E. rostratum* MD of 6.97–7.63 cm and 4.96–5.83 cm, respectively (Table 5). No significant *E. rostratum* MD differences recorded between the CMA and CA medium. The *E. rostratum* MD scored on PDA and MEA showed significant differences under different light cycles. On PDA, the highest *E. rostratum* MD was recorded under complete darkness (T4) followed by T1 (12 h fluorescent light + 12 h black light), T3 (12 h black light + 12 h complete darkness) and T2 (12 h fluorescent light + 12 h complete darkness). Meanwhile, MEA recorded the least *E. rostratum* MD in all light cycles. The highest *E. rostratum* MD on MEA was recorded in T1,significantly different from the other treatments. T3 showed the smallest *E. rostratum* MD (4.96 cm), much lower than the *E. rostratum* MD in CMA and CA (Table 3).

**Table 3:** Effect of growth medium and light cycle on *Exserohilum rostratum* mycelium diameter and sporulation activity.

Media	Treatment	Diameter (cm)	No. of spores (× 10^6^/ml)
Potato Dextrose Agar (PDA)	T1	7.12 ± 0.062b	5.00 ± 0.048b
	T2	6.97 ± 0.065b	4.67 ± 0.050b
	T3	7.05 ± 0.053b	2.50 ± 0.043c
	T4	7.63 ± 0.056a	15.50 ± 0.057a
Corn Meal Agar (CMA)*	T1	7.70 ± 0.058a	19.00 ± 0.060b
	T2	7.70 ± 0.055a	21.67 ± 0.062a
	T3	7.70 ± 0.051a	13.83 ± 0.051c
	T4	7.70 ± 0.052a	21.50 ± 0.053a
Czapex-dox agar (CA)*	T1	7.70 ± 0.063a	12.00 ± 0.058a
	T2	7.70 ± 0.069a	12.67 ± 0.051a
	T3	7.70 ± 0.054a	3.50 ± 0.059c
	T4	7.70 ± 0.059a	7.00 ± 0.061b
Malt Extract Agar (MEA)	T1	5.83 ± 0.071a	0.67 ± 0.044ab
	T2	5.40 ± 0.069b	0.33 ± 0.052b
	T3	4.96 ± 0.064c	1.00 ± 0.061ab
	T4	5.01 ± 0.059bc	1.33 ± 0.040a

*Plates subjected to sporulation activity determination.

All values are expressed as average mean ± standard deviation of three independent experiments. Treatments are denoted as following: T1; 12 h fluorescent light + 12 h black light, T2; 12 h fluorescent light + 12 h complete darkness, T3; 12 h black light + 12 h complete darkness , and T4; 24 h darkness.

*Means in column with different letter are significantly different at *p* $\le$ 0.05 level according to Fisher’s least significant difference (LSD).

Sporulation activity of *E. rostratum* (measured by the spore count) was the highest on CMA at 18.88 × 10^6^ spores/ml. The sporulation activity of *E. rostratum* under various medium was significantly different; the lowest activity was recorded on MEA followed by CA and PDA. CMA produced the highest number of spores (T2 with and T4), significantly higher than the others. In CA supplemented *E. rostratum*, the highest spore production was observed in T1 and T2: no significant differences in sporulation activity. The *E. rostratum* on PDA produced the lowest number of spores; T3 (2.50 × 10^6^ spores/ml) was significantly different to T1 and T2. In MEA supplemented *E. rostratum*, the highest spore production was observed in T4 while the lowest was recorded by T2(Table 4).

**Table 4:** Effect of growth medium and light cycle on *Exserohilum rostratum*, fungal pathogen.

Media/treatment	Diameter (cm)	No. of spore (× 10^6^)
Potato dextrose agar (PDA)	7.19b	6.92c
Corn meal agar (CMA)	7.70a	18.88a
Czapex-dox agar (CA)	7.70a	8.79b
Malt extract agar (MEA)	5.30c	0.83d
T1	7.09a	9.17b
T2	6.94bc	9.71b
T3	6.85c	5.21c
T4	7.01ab	11.33a
LSD (0.05)	0.14	0.81

Treatments are denoted as CMA supplemented *E. rostratum* under the following light cycles: T1; 12 h fluorescent light + 12 h black light, T2; 12 h fluorescent light + 12 h complete darkness, T3; 12 h black light + 12 h complete darkness , and T4; 24 h darkness.

* Means in column with different alphabets are significantly different at *p* $\le$ 0.05, Fisher’s least significant difference (LSD).

### Effect of silicon fertilization on brown spot disease severity

There were no significant differences in brown spot index (BSI) between the two rice varieties tested. At the rice harvest stage, the BSI values of MR219 and MR253 rice varieties were 33.11% and 31.97%, respectively. However, on an individual rice variety-specific evaluation, significant differences were observed among treatments. For MR219 plants, the lowest BSI was recorded in T5 (CaSiO_3_ + Mn) (29.94%) followed by T3 (RHA + Mn) (30.50%), T2 (RHA) (31.37%) and T4 (CaSiO_3_) (32.25%) while for MR253 plants, the lowest BSI was recorded by T3 (RHA + Mn) (28.20%) followed by T5 (CaSiO_3_ + Mn) (29.11%), T2 (RHA) (30.70%) and T4 (CaSiO_3_) (30.98%). In both rice varieties, application of Mn lowered BSI values though with an insignificant difference compared to treatments without Mn application. In MR219 plants, the application of RHA or CaSiO_3_ with Mn lowered the BSI by 0.87 and 2.31, respectively. Likewise in MR253 plants, similar applications lowered BSI by 2.50 and 1.87, respectively (Table 5).

**Table 5:** Brown spot index (BSI) of MR219 and MR253 rice varieties cultivated under different treatment conditions.

Variety	Treatment	GY (%)
MR219	T1 (Control)	41.51 ± 3.2a
	T2 (RHA)	31.37 ± 2.7b
	T3 (RHA + Mn)	30.50 ± 3.2b
	T4 (CaSiO_3_)	32.25 ± 2.9b
	T5 (CaSiO_3_ + Mn)	29.94 ± 2.3b
MR253	T1 (Control)	40.87 ± 3.1a
	T2 (RHA)	30.70 ± 2.8b
	T3 (RHA + Mn)	28.20 ± 2.5b
	T4 (CaSiO_3_)	30.98 ± 2.7b
	T5 (CaSiO_3_ + Mn)	29.11 ± 3.1b
Mean comparison	MR219	33.11 ± 2.1a
between variety	MR253	31.97 ± 2.8a

Treatment combinations are represented as following: T1 = control (standard fertilizer, NPK only), T2 = NPK + rice husk ash (RHA), T3 = NPK + RHA + manganese (Mn), T4 = NPK + calcium silicate (CaSiO_3_), T5 = NPK + CaSiO_3_ + Mn. All values are presented as average means ± standard deviations of three independent experiments. Values in the column with different alphabets are significantly different at *p* ≤ 0.05 Fisher’s least significant difference (LSD).

### Effect of silicon fertilization on grain yield

The grain yield was expressed in percentage, taking into account parameters such as filled grains and discolouration-free grains. Both MR219 and MR253 displayed a similar trend. The Si incorporated treatments, in combination with and without Mn (T2, T3, T4 and T5) showed significantly higher grain yield (GY) in comparison to the non-treated, Si-free plants (T1). In the MR219 rice variety, RHA in combination with Mn (T3) showed the highest GY at 79.54%. The MR253 variety, in contrast, showed that the CaSiO_3_ in combination with Mn-treated plants (T5) showed the highest GY at 78.83%. Although rice plants treated with Si in combination with Mn showed higher GY in both MR219 and MR253 varieties, nevertheless, the yield was insignificant (Table 6).

**Table 6:** Grain yield (GY)% of MR219 and MR253 rice varieties (harvest stage) cultivated under different treatment conditions.

Variety	Treatment	GY (%)
MR219	T1 (Control)	61.23 ± 5.8a
	T2 (RHA)	71.31 ± 4.5b
	T3 (RHA + Mn)	79.54 ± 8.8b
	T4 (CaSiO_3_)	75.83 ± 6.4b
	T5 (CaSiO_3_ + Mn)	76.19 ± 6.3b
MR253	T1 (Control)	57.61 ± 7.1a
	T2 (RHA)	73.66 ± 8.5b
	T3 (RHA + Mn)	77.21 ± 6.5b
	T4 (CaSiO_3_)	74.69 ± 7.7b
	T5 (CaSiO_3_ + Mn)	78.83 ± 7.1b

Treatment combinations are represented as following: T1 = control (standard fertilizer, NPK only), T2 = NPK + rice husk ash (RHA), T3 = NPK + RHA + manganese (Mn), T4 = NPK + calcium silicate (CaSiO_3_), T5 = NPK + CaSiO_3_ + Mn. All values are presented as average means ± standard deviations. Values in the same column with different alphabets are significantly different at *p* ≤ 0.05 Fisher’s least significant difference (LSD).

## Discussion

In Malaysia, rice is an important staple food for about 32 million people of its population. This food crop contributes to 26% of the total calorie intake of an average Malaysian at a cost of between RM13 to RM73 per month per household^2^. According to National Agro-Food Policy (2011–2020), the rice industry in Malaysia has displayed a lagging pattern in terms of productivity in recent years. Despite various measures introduced for productivity enhancement, to date, Malaysia remains a mere net importer with a self-sufficiency level of 70%. The country is still struggling to secure enough rice to feed its people whilst her neighbouring counterparts such as Thailand, Vietnam, China, India and Bangladesh are experiencing a bountiful production that exceeds their total consumption^2^.

There are 10 key granaries in Peninsular Malaysia, namely, Muda Agricultural Development Authority (MADA) located in Northern Peninsular which contributes to about 40% of the national rice production (biggest rice bowl area in Malaysia), Kemubu Agricultural Development Authority (KADA), Integrated Agricultural Development Area (IADA) Barat Laut Selangor, IADA Pulau Pinang, IADA Seberang Perak, IADA Krian, IADA Ketara, IADA Pekan, IADA Rompin and IADA Kemasin Semarak. These granaries are subjected to different farming practices, geographical conditions, soil nutrients and fertility, and climate. The national average rice yield amounts up to 4.0 tonne/ha but at high-performing granaries it exceeds 5.0 tonne/ha, while in low-performing granaries the yield is below 3.0 tonne/ha. In this study, the five different granaries selected for brown spot disease pathogen isolation are comprised of high (Sungai Jagong-MADA, Bumbong Lima-IADA Pulau Pinang, Sungai Besar-IADA Barat Laut Selangor) and low performing granaries (Melor-KADA, Krian-IADA Kerian)^2^. Both morphological and molecular analyses identified *E. rostratum* and *B. oryzae* species as the causal agents of rice BS in Peninsular Malaysia.

Rice fungal diseases are most prevalent in Si and nutrient depleted soils in Malaysia^73^. In others, rice Si fertilization was shown to significantly reduce BS disease severity^74,75^. As the source of Si, both RHA and CaSiO_3_ were evaluated with and without the combination of Mn, a micronutrient essential in plant development and defense system^76^. The Mn absorption by plants aids the activation of key enzymes involved in the phenylpropanoid pathway which governs the biosynthesis of phenolic polymers that are resistant to enzymatic degradation by fungal pathogens^62^. In this study, rice plants amededed with Si (with and without Mn) at various treatment combinations showed no significant differences in BSI and rice grain yield (GY). The results were in agreement with Zanão et al.^77^ who reported that rice plants subjected to Si fertilization in presence of Mn show no apparent differences on disease severity. It is hypothesized that foliar application of Mn might have been more effective in reducing BSI as it directly targets the leaves where BS is observed on rice.

Rice husk (RH) is a hard and protective covering that envelopes the grain or brown rice. It constitutes cellulose (50%), lignin (25–30%), silica (14–20%) and moisture (10–15%). During a typical milling process, the grains are separated from the husks, which then results to agricultural waste. On average, milling converts a harvested paddy plant into rice (78%), rice husk (20%) and the residue which is the remainder lost throughout the mechanical process. With the growing population, the world rice producing capacity has been increasing over recent years^78^ and in major rice-producing countries, rice husk agro-waste is either recycled for low-value applications or disposed of in landfills and open fields through open burning. Burning RH yields rice husk ash (RHA), with 83–90% silica in the amorphous state. RHA enriched with amorphous Si holds good potential for rice biofertilization.

Rice is a classic Si-accumulator. The plant accumulates Si beneficial element at a broad level that sometimes it may exceed 10% of its dry weight^23^. Si is absorbed by plant roots in the form of silicic acid which is then transported to the shoot where it gets concentrated during loss of water (transpiration). The absorbed silicic acid polymerizes into colloidal silicic acid and finally develops into silica gel^79,80^. Si fertilization in rice farming improves resistance against lodging and agronomic traits^80–82^, ameliorates troubled soiled conditions such as high metal toxicity and acidity^83–85^ and enhance host resistance against pest and pathogens^86^. The utilization of RHA as a form of Si fertilization is poorly unleashed, especially among the Southeast Asian rice net importer countries such as Malaysia and Indonesia. As such, rice farming in Malaysia is currently subjected to pesticide and fertilizer input solely. The application of Si fertilizer as part of rice farming practice is not reported in Malaysia. Our findings revealed that the application of RHA was comparable to CaSiO_3_, a chemical fertilizer commonly used in rice farming as both treatments showed no significant difference between them in BSI against BS caused by *E. rostratum*. Further, Si fertilization positively impacts the grain yield as the yield in the Si-treated plants was significantly higher than the non-Si-treated plants^87^.

The present findings enhanced our understanding of BS disease causal agents in rice granaries across Peninsular Malaysia. The current findings showed that rice BS in Peninsular Malaysia is caused by both *E. rostratum* and *B. oryzae* fungal pathogens. Irrespective of each granary’s yield potential and geographical conditions, *E. rostratum* was found to be the predominant BS causal agent compared to *B. oryzae*. The *E. rostratum* was first reported as the causal agent of rice BS in Malaysia in 2015^15^ whilst most previously reported studies have associated the occurrence of BS to *Bipolaris* genus solely^16^.

In Malaysia, farmers’ primary strategy toward disease management is through the application of fungicides and pesticides. Having been exposed to these chemicals, the native pathogen population may have responded to selection pressure and resulted in *E. rostratum* evolution. The present study identified two different species as the causal agents of BS in Peninsular Malaysia. Nevertheless, future research should probe for BS causal agents at multiple growing seasons within the same rice granaries to understand the time-scale BS pathogen species diversity evolvement. Further, we suggest that Si-fertilization should be incorporated using RHA as a renewable source for a sustainable rice production system.
